# Newcastle Disease Virus Displaying an Ectodomain of Middle East Respiratory Syndrome Coronavirus Spike Protein Elicited Robust Humoral and Cellular Immunity in Mice

**DOI:** 10.3390/vaccines13010002

**Published:** 2024-12-24

**Authors:** Jaturawitt Prasopsiri, Kanjana Srisutthisamphan, Benjamas Liwnaree, Juggragarn Jengarn, Jarin Kramyu, Payuda Hansoongnern, Papon Muangsanit, Nathiphat Tanwattana, Challika Kaewborisuth, Suttipun Sungsuwan, Anan Jongkaewwattana, Nanchaya Wanasen

**Affiliations:** National Center for Genetic Engineering and Biotechnology, National Science and Technology Development Agency, Khlong Nueng, Khlong Luang, Pathum Thani 12120, Thailand; jaturawitt.pra@ncr.nstda.or.th (J.P.); kanjana.sri@biotec.or.th (K.S.); benjamas.chu@biotec.or.th (B.L.); juggragarn.jen@biotec.or.th (J.J.); jarin@biotec.or.th (J.K.); payuda.han@ncr.nstda.or.th (P.H.); papon.mua@biotec.or.th (P.M.); nathiphat.tan@ncr.nstda.or.th (N.T.); challika.kae@biotec.or.th (C.K.); suttipun.sun@biotec.or.th (S.S.); anan.jon@biotec.or.th (A.J.)

**Keywords:** Newcastle disease virus, MERS-CoV, vaccine, spike, neutralizing antibody, mouse model

## Abstract

Background: Middle East Respiratory Syndrome Coronavirus (MERS-CoV) causes severe respiratory illness in humans and currently lacks an approved vaccine. The Newcastle disease virus (NDV) vector is a well-established, safe, and effective platform for vaccine development. With recent advancements in stabilizing coronavirus spike proteins to enhance their antigenicity, this study aimed to determine whether modifications to the MERS-CoV spike protein could improve its presentation on NDV particles, allowing the resulting virus to be used as an inactivated vaccine. Methods: We codon-optimized the gene encoding the ectodomain of the MERS-CoV spike protein and incorporated modifications at the S1/S2 and S2’ cleavage sites, along with a proline substitution at residues V1060-L1061. This modified spike gene was inserted into the NDV genome to create the NDV-S_MERS_ virus. After purification and inactivation, the vaccine’s immunogenicity was assessed in mice. Results: Mice immunized with the inactivated NDV-S_MERS_ vaccine developed robust anti-spike IgGs, neutralizing antibodies, and cellular immune responses. The study demonstrated that modifications to the MERS-CoV spike protein were essential for its effective presentation on NDV particles. Additionally, the spike gene insert remained stable through five egg passages, confirming the vector’s stability. Conclusions: Engineering the MERS-CoV spike protein is crucial for its successful display on NDV particles. The strong immune responses elicited by the NDV-S_MERS_ vaccine in mice highlight that NDV is a promising, safe, and effective platform for MERS-CoV vaccination.

## 1. Introduction

Middle East Respiratory Syndrome (MERS) is a severe respiratory disease caused by the MERS-coronavirus (MERS-CoV). MERS-CoV is a zoonotic virus typically found in its natural host, *Camelus dromedarius* (dromedary camels). However, the virus occasionally spills over into humans, where it can cause communicable severe respiratory illness [1]. In 2015, an outbreak of MERS occurred in Korea from a man who traveled to the Middle East region. The outbreak led to 186 confirmed cases that included 38 deaths [2]. Significant effort has been dedicated to the development of MERS vaccines; however, as of now, no vaccines have been approved for human use.

Various vaccine platforms have been explored for MERS vaccine development including ChAdOx1, MVA, and DNA vaccines [3]. Newcastle disease virus (NDV) is another promising vaccine platform for MERS. While the NDV-based MERS vaccine has been tested only in animals such as mice and camels [4], the NDV-based vaccine for severe acute respiratory syndrome coronavirus 2 (SARS-CoV-2) has been shown to be safe and capable of eliciting robust immune responses in humans [5,6].

One of the factors attributed to the success of the NDV-based COVID-19 vaccine was the strategic design of SARS-CoV-2 spike protein to maintain its immunogenic prefusion form. Hsieh et al. demonstrated that 6 proline substitutions (called HexaPro) in the S2 region of SARS-CoV-2 spike protein drastically increased the stability of the prefusion form of the protein [7]. Proline substitutions were shown to similarly stabilize MERS-CoV spike (S_MERS_) protein. Pallesen et al. demonstrated that two proline substitutions at residues V1060 and L1061 (called 2P mutations) retained the soluble recombinant S_MERS_ ectodomain in its prefusion form [8]. To our knowledge, 2P-mutated S_MERS_ ectodomain has not been employed in NDV-based vaccine development.

In this study, we evaluated whether NDV carrying S_MERS_ ectodomain (NDV-S_MERS_) could serve as an effective MERS vaccine. We found that in order to achieve surface S_MERS_ expression, S1/S2, S2’ cleavage site mutations, together with the 2P mutation were needed. We also demonstrated that the NDV-based vaccine, both in inactivated and live vaccine formats, efficiently elicited strong MERS-CoV-specific IgGs, neutralizing antibodies, and cellular immune responses in mice. Furthermore, it was shown that the *S_MERS_* gene insert was intact after 5 egg-passages, providing evidence that NDV is a promising platform for developing a MERS vaccine.

## 2. Materials and Methods

### 2.1. S_MERS_ Codon Optimization and the Analyses of Codon Contents

The native S_MERS_ sequence was retrieved from Genbank (accession number KT026454). The mammalian-codon optimization was generated using the iCodon program (www.iCodon.org (accessed on 1 February 2023) [9]), while the chicken-codon optimized sequence was generated using GenSmart Codon Optimization tool from www.genscript.com. The codon content analyses, including Codon Adaptation Index (CAI), GC Content at the Third Position of Synonymous Codons (GC3) and Codon Pair Scores (CPS) of the native and the codon optimized *S_MERS_* sequences were calculated using simple sequence editor (SSE) software version 1.4 [10].

### 2.2. NDV Infectious Clone Construction and Rescue

Synthesized DNA fragments (Synbio Technologies, Monmouth Junction, NJ, USA) corresponding to the genomic sequence of NDV La Sota C5 strain (GenBank number KC844235.1) with modifications needed to facilitate convenient cloning were assembled using an Infusion cloning technique (Takara Bio, San Jose, CA, USA) into a pCI plasmid (also synthesized by Synbio Technologies) already containing the regulatory features needed for NDV RNA synthesis. Initially, the construct was made of NDV La Sota C5 genome containing, between *P* and *M* genes, an open reading frame of *mCherry* gene flanked by MluI and NotI restriction enzyme sites, and NDV gene-stop and gene-start signals necessary for a transcription of the mRNA insert. When necessary, this construct was digested with MluI and NotI, and subsequently used for inserting *S_MERS_* gene into the NDV genome.

The constructs containing *mCherry* and *S_MERS_* gene were used to generate NDV-mCherry and NDV-S_MERS_ viruses, respectively. Briefly, BHK-21/WI-2 (Kerafast, Shirley, MA, USA) were transfected with plasmids pCI.NDV-mCherry or pCI.NDV-S_MERS_ (1 µg) along with the following supporting plasmids: pCAGGS.T7 polymerase (1 µg), pCAGGS.NP (0.4 µg), pCAGGS.P (0.2 µg), and pCAGGS.L (1 µg). The plasmids were then mixed with Lipofectamine 3000 Reagents (Thermo Fisher Scientific, Waltham, MA, USA) using the manufacturer suggested protocol. The transfection mixture was subsequently added dropwise to BHK-21/WI-2 cells (400,000 cells in 6-well plates). The culture plates were then rocked for 15 min at 37 °C, then added with 1 mL Opti-MEM (Thermo Fisher Scientific), which was then replaced with fresh medium after 6 h. The cells were incubated at 37 °C for 24 h. Following incubation, the transfected cells were co-cultured with DF-1 cells in Opti-MEM containing 0.1% TrypLE (Thermo Fisher Scientific). The cells were left undisturbed for 72 h. NDV-mCherry-infected cells were observed for red fluorescence under a fluorescence microscope, and NDV-S_MERS_-infected cells were detected by immunofluorescence staining. Culture media and cells were harvested for further virus propagation in embryonated chicken eggs.

### 2.3. Virus Amplification

Specific pathogen free embryonated chicken eggs were obtained from Thai SPF Co, Ltd. (Nakhon Nayok, Thailand). Eggs were incubated at 37 °C, and automatically turned every hour for 9 days. Eggs containing viable embryos were selected for inoculation with 100 µL cell culture medium from the virus-rescue cultures. Following a 72-h incubation at 37 °C, allantoic fluid was harvested. After a centrifugation at 3000× *g* for 30 min at 4 °C to remove tissue and cell debris, the allantoic fluid was subjected to viral titration via a hemagglutination (HA) assay using 0.7% chicken red blood cells.

### 2.4. Virus Ultracentrifugation

Allantoic fluid samples that had positive titers in an HA assay were combined and subjected to ultracentrifugation at 50,000× *g* for 2 h at 4 °C. The resulting virus pellet was then resuspended in 0.01 M Tris-NaCl buffer (pH 7.2) and subsequently purified through 20% and 50% sucrose (*w*/*v*) layers by ultracentrifuging at 50,000× *g* for 2 h at 4 °C. The resulting virus layer was collected and resuspended in PBS before another centrifugation step at 50,000× *g* for 2 h at 4 °C to remove sucrose from the sample. The resulting pellet was dissolved in PBS and filtered through a 0.22 µm filter. The purified virus was concentrated by reducing its volume through an Amicon Ultra NMW0 50 kDa filter (MilliporeSigma, Burlington, MA, USA).

### 2.5. Immunofluorescence Staining

Uninfected and NDV-infected DF-1 cells were fixed with 80% ice-cold acetone or 4% formaldehyde for 20 min. After 3 washes with PBS, samples were incubated with a blocking buffer (1% BSA and 10% FBS in PBS) for 1 h, followed by an incubation with mouse anti-MERS-CoV S1 monoclonal antibody (MAB10707, R&D system’s, Minneapolis, MN, USA) for 1 h at room temperature. After 3 washes with 0.1% PBS-Tween (PBST, 1× PBS with 0.1% Tween-20), the cells were incubated with AlexaFlour-488 conjugated goat anti-mouse IgGs (A11004, Thermo Fisher Scientific) for anti-MERS-CoV S1 detection. The samples were washed 3 times with 0.1% PBST, then the stained cells were visualized under a fluorescence microscope.

### 2.6. Virus Inactivation

Binary ethylenimine (BEI) at 0.1 M concentration was freshly prepared (Sigma Aldrich, St. Louis, MO, USA). BEI solution was added to the purified virus samples to a final concentration of 1 mM. The samples were then incubated at 37 °C for 24 h. BEI was then neutralized with 10 mM sodium thiosulphate for 2 h at room temperature.

### 2.7. Transmission Electron Microscopy (TEM)

Ultracentrifuged virus samples (10 µL) were adsorbed onto a CF-200-CU carbon support films (Electron Microscope Sciences, Hatfield, PA, USA) for 10 min. After one wash with water, the films were incubated with blocking buffer (Electron Microscope Sciences) for 30 min, followed by 6 washes, 3 min each, with a wash buffer (PBS containing 1% BSA). The samples were then incubated with rabbit anti-MERS-CoV S1 antibody (Sino Biological, Beijing, China) for 60 min. After 6 washes, the samples were incubated with 10 nm gold-conjugated goat anti-rabbit antibody (Electron Microscope Sciences) for 60 min. After washing 6 times, the samples were fixed with 2% glutaraldehyde diluted in the wash buffer, followed by a staining with 3% phosphotungstic acid. The films were air-dried, then observed under a Hitachi HT7700 transmission electron microscope at 80.0 kV (Hitachi High-Technologies, Tokyo, Japan).

### 2.8. S_MERS_-Specific Sandwich ELISA

ELISA plates were coated with 0.5 µg/mL monoclonal mouse anti-MERS-CoV S1 (MAB10707, R&D Systems) overnight. After 3 washes with PBST, blocking buffer (1% BSA and 1% skim milk in PBST) was added to each well. Following a 1-h incubation at 37 °C, the plates were washed and added with 100 µL of purified virus samples, or 0.005, 0.01, 0.02, 0.04 and 0.08 µg/mL recombinant S_MERS_ protein standards (R&D system’s). The plates were incubated for 2 h at 37 °C, then washed, and subsequently added with rabbit anti-S_MERS_ (40069-R723, Sino Biological). Plates were incubated for 1 h at 37 °C, then subsequently added with HRP-conjugated goat anti-mouse IgGs. After a 1-h incubation, the plates were washed and subsequently added with TMB substrate. After 15 min, 2 N H_2_SO_4_ were added and the absorbance was measured at 450 nanometers with an ELISA plate reader (EnSight Multimode Plate Reader, PerkinElmer, Shelton, CT, USA). The concentrations of S_MERS_ protein in the purified virus samples were calculated based on the standard curve of the recombinant S_MERS_ protein.

### 2.9. Mouse Immunization

Groups of 5 ten-week old Balb/cAJc mice (Nomura Siam International, Bangkok, Thailand) were injected twice intramuscularly, 3 weeks apart, with 50 µL of the following: (1) PBS (2) inactivated NDV-mCherry (3) recombinant S_MERS_ (4) a low dose inactivated NDV-S_MERS_-S2’ (5) a high dose inactivated NDV-S_MERS_-S2’ or (6) live NDV-S_MERS_-S2’. All the vaccine regimens, except for the live NDV-S_MERS_-S2’, were prepared as a mixture with Addavax adjuvant (InvivoGen, San Diego, CA, USA). The low and high doses of inactivated NDV-S_MERS_-S2’ contained 100 and 250 ng of S_MERS_ as measured by S_MERS_-specific sandwich ELISA, respectively. The concentration of the high dose inactivated NDV-S_MERS_-S2’ was 3.9x10^6^ TCID50/mL. This concentration was used to prepare an equivalent dose of the live NDV-S_MERS_-S2’. In-house recombinant S_MERS_ was used at 250 ng/dose. Two weeks after the second immunization, mice were sacrificed for blood and splenocyte collections.

### 2.10. ELISPOT

Mouse spleens were collected and ground through 70 µm cell strainers. The collected cell suspensions were subjected to red blood cell lysis with a red blood cell lysis buffer (PAN Biotech, Aidenbach, Germany). The resulting splenocytes were then placed in ELISPOT plates coated with anti-mouse IFN-γ antibodies (CTL, Cleveland, OH, USA) at 500,000 cells per well in the presence of 1 µg/mL S_MERS_ overlapping peptides (Mimotopes, Victoria, Australia). After a 24-h incubation at 37 °C, the cells were washed off and the ELISPOT plates were developed according to the manufacturer’s protocol. The numbers of dots per well were analyzed using ImmunoSpot software (ImmunoSpot 7.0.9.4 Professional Analyzer DC, CTL) on a CTL ImmunoSpot S6 Universal Analyzer (CTL).

### 2.11. S_MERS_-Specific IgG ELISA

Recombinant S_MERS_ protein was produced in-house from 293A cells (Expi293™Expression System, Thermo Fisher Scientific) transfected with pSecTag2A.S_MERS_ designed to express secreted S_MERS_ ectodomain containing S1/S2, S2’ and 2P mutations. The S_MERS_ protein diluted in pH 9.6 carbonate buffer was used to coat ELISA plates overnight at 4 °C. The next day, the ELISA plates were added with PBS containing 0.1% Tween-20, 0.5% BSA and 0.5% skim milk, and subsequently incubated at 37 °C for 2 h. Mouse serum samples that were 10-fold serially diluted in 1× PBS containing 0.1% Tween-20, 0.5% BSA and 0.25% skim milk were then added. The plates were incubated at 37 °C for 2 h, and subsequently added with 1:4000 diluted HRP-conjugated goat anti-mouse IgGs. The plates were then incubated at 37 °C for 1 h before TMB substrate was added. After 15 min incubation, 2 N H_2_SO_4_ was added and the absorbance at 450 nm was measured by an ELISA plate reader (EnSight Multimode Plate Reader, PerkinElmer).

### 2.12. Pseudotyped Virus Neutralization Assay

Pseudotyped virus displaying S_MERS_ protein was generated by transfecting HEK293T/17 cells with (1) pCSFL plasmid containing lentivirus genome with a luciferase reporter gene insert; (2) pCMVΔR8.91 expressing Gag and Pol proteins that allowed the virus to replicate in cultured cells; (3) pCAGGS.S_MERS_ that expressed codon-optimized, unmutated S_MERS_ protein. At 72 h post transfection, pseudotyped virus-S_MERS_ (PV-S_MERS_) was harvested. The PV-S_MERS_ was then 10-fold serially diluted and its optimal working concentration was determined by transducing the PV-S_MERS_ into HEK-293T/17-DPP4 cells (generated in-house). The dilution that yielded luminescent signals around 100,000 units was used for neutralizing assays. Briefly, 50 µL of 2-fold serially diluted heat-inactivated mouse sera was incubated with 50 µL of diluted PV-S_MERS_ in 96-well plates. After a 1 h incubation at 37 °C, HEK-293T/17-DPP4 cells in suspension (5 × 10^4^ cells/well) were mixed with the prepared serum-virus mixtures. Then after a 48-h incubation at 37 °C, cell culture medium was removed, and lysis buffer mixed with luciferase enzyme substrate was added. Luminescence signals were measured with BioTek Synergy HTX Multimode Reader (Agilent, Santa Clara, CA, USA). Pseudotyped virus neutralization-50 (PVNT-50) was calculated on a GraphPad Prism 9.0.0 (121) (GraphPad Software, Boston, MA, USA) using nonlinear regression curve fit [11]. PVNT-50 was documented as the dilution of sera that reduced luminescence signals to 50% of the no serum control wells.

### 2.13. Statistical Analyses

All statistical analyses of the data were performed using the built-in functions of GraphPad Prism 9.0.0 (GraphPad Software, Boston, MA, USA). The Shapiro-Wilk test (alpha = 0.05) was used to ensure the normal distribution of the data sets. Data sets that passed the Shapiro-Wilk test (S protein ELISA and ELISPOT data sets) were subsequently analyzed for significant differences using one-way ANOVA. Data sets that did not pass the Shapiro-Wilk test (PVNT50 and anti-S IgG ELISA data sets) were analyzed for significant differences using the Kruskal-Wallis test. Tukey’s multiple comparisons test was used to determine the significant differences in S protein presentation among all groups. Dunnett’s multiple comparisons post-test was used to determine the significant differences in immune responses between the vaccinated groups to the NDV-mCh vaccinated controls.

## 3. Results

### 3.1. High Levels of S_MERS_ Expression Required Codon Optimization

Efficient antigen presentation is one of the desired qualities of a vaccine candidate. A previous study by Wu et al. showed that the sequences of SARS-CoV2 isolates are deoptimized for protein expression in human cells, and that codon optimization significantly enhanced SARS-CoV2 spike expression in HEK-293T cells [12]. To ensure that our vaccine constructs had an optimal S_MERS_ sequence for protein expression in host cells, we utilized iCodon (www.iCodon.org (accessed on 14 February 2023)) and GenSmart Optimization tools (www.genscript.com (accessed on 1 March 2023)) to generate mammalian- and chicken-codon optimized S_MERS_ sequences.

**Figure 1:**
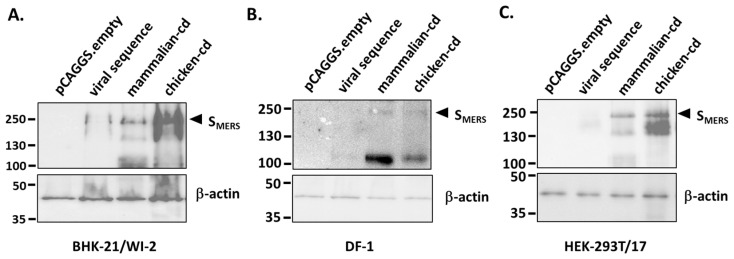
Codon optimization strongly enhanced S_MERS_ expression. pCAGGS.empty, or pCAGGS plasmids carrying either the native viral sequence (viral sequence), mammalian-codon optimized sequence (mammalian-cd), or chicken-codon optimized sequence (chicken-cd) were transfected, 1 µg each, into (**A**) BHK-21/WI-2, (**B**) DF-1 cells, or (**C**) HEK-293T/17 cells. At 72 h post transfection, cell lysates were collected and S_MERS_ expression was measured by Western blot using rabbit anti-S2 antibody as the primary antibody.

To validate that the codon optimization improved S_MERS_ protein expression, the native viral and the codon optimized sequences were cloned into an expression plasmid and used to transfect BHK-21/WI-2 and DF-1 cells, the two cell lines used in generation of recombinant NDVs. As shown in Figure 1, we observed that S_MERS_ protein derived from the native viral sequence was barely detectable on a Western blot, but the mammalian- or chicken-codon optimized sequence yielded clear protein bands corresponding to the full-length S_MERS_ protein. Note that the protein bands in DF-1 cells were fainter than those from transfected BHK-21/WI-2 cells, possibly due to a lower transfection efficiency of DF-1 cells. We also used HEK-293T/17 cells, another mammalian cell that could also be used to rescue NDV virus, to confirm our findings. The results in Figure 1C, showed that the codon-optimization enhanced S_MERS_ expression in HEK-293T/17 cells. Together, the data suggested that codon-optimization is crucial for achieving high levels of S_MERS_ expression in host cells.

To identify factors contributing to the improved protein expression, a sequence analyzer, SSE software [10], was used to calculate essential parameters reported to impact levels of protein expression. A codon adaptation index (CAI) [13], which indicates the frequency of preferred codons in each host species, was calculated. As shown in Table 1, the CAI analysis showed that the codon-optimized sequences, as compared to the native viral sequence, contained a higher frequency of preferred codon usage for the corresponding hosts. The codon optimized sequences also had higher GC content at the third position of synonymous codons (GC3) scores, known to be positively correlated with protein expression levels [14]. However, the codon pair scores (CPS), which indicate the frequency of preferred codon pair [15,16], suggested that both mammalian- and chicken-codon optimized sequences contained fewer preferred codon pairs (as indicated by the negative CPS values) compared to the viral sequence. Thus, the higher protein expression of the codon-optimized sequences was less likely affected by a codon pair bias, but rather, positively impacted by a higher frequency of preferred single codons in the sequences.

### 3.2. Cleavage Site Modifications Were Required for Surface Expression of S_MERS_

The S_MERS_ protein is a viral surface glycoprotein responsible for binding to host receptors and facilitating viral-host membrane fusion. Therefore, its cleavage is essential for exposing the fusion peptide required for membrane fusion. However, an uncleaved, full-length ectodomain of S_MERS_ may serve as a better antigen for vaccines. Consequently, several modifications to the S_MERS_ protein may be necessary when incorporating it into an NDV vector.

Similar to other coronaviruses, S_MERS_ contains protease cleavage sites at S1/S2 and S2’ positions. The S1/S2 position (748-RSVR-751) has been reported to be cleaved by endogenous proteases, including furin, during biosynthesis in the virus-producing cells. Meanwhile, the S2’ cleavage site (884 RSAR 887), located upstream of the fusion peptide, was reported to be cleaved during viral entry by furin, cathepsin L, or TMPRSS2, depending on the cell type [17,18]. Single or double arginine mutations at the S1/S2 position have been shown to result in the secretion of uncleaved S_MERS_ product [19,20], while arginine mutations at the S2’ position lead to the absence of a small 65 kDa S2’ cleaved product [20]. Additionally, proline substitutions at residues V1060 and L1061 are essential for maintaining S_MERS_ in a stable prefusion form [8].

**Figure 2:**
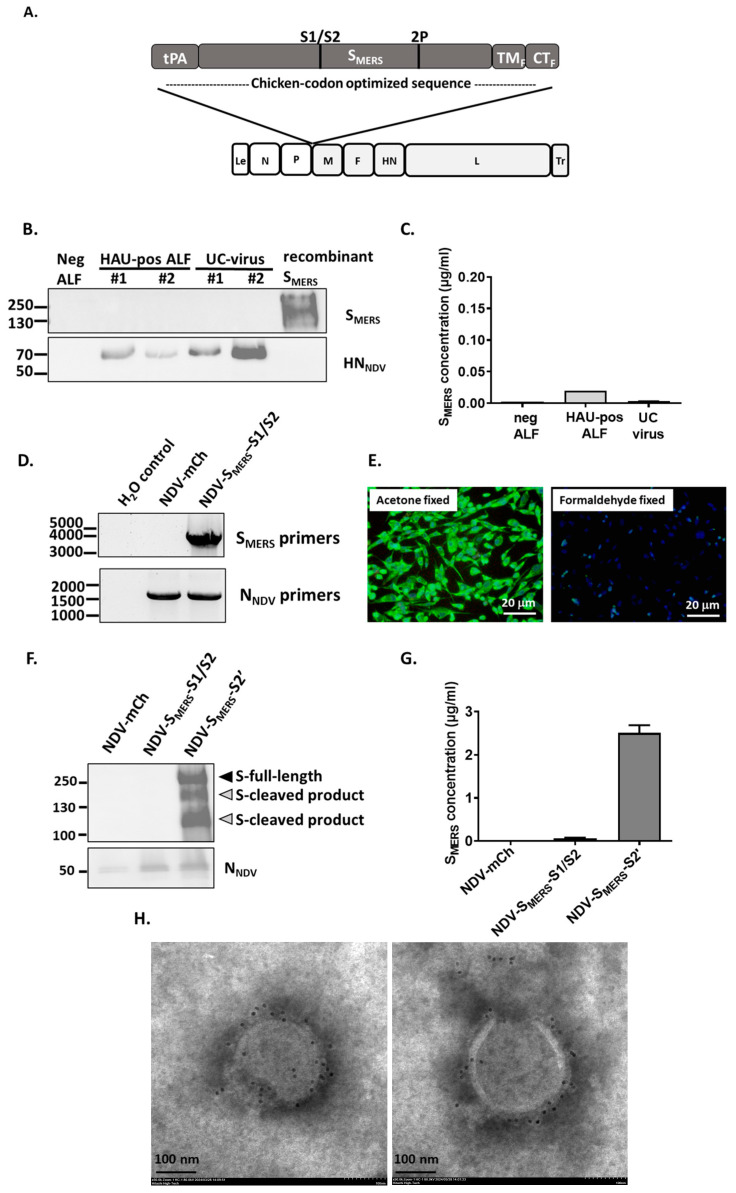
Mutations at S1/S2, S2’ positions were needed for S_MERS_ to be expressed on the surface NDV particles. (**A**) The genomic arrangement of NDV-S_MERS_-S1/S2_._ Chicken-codon optimized S_MERS_ with S1/S2 cleavage site mutation, 2P mutation, tPA secretion peptide, transmembrane domain (TM_F_) and cytoplasmic tail (CT_F_) of NDV F protein was inserted between *P* and *M* genes of NDV genome. (**B**) Allantoic fluid (ALF) harvested from embryonated chicken eggs inoculated with NDV-S_MERS_-S1/S2, and the ultracentrifuged virus samples (UC-virus) were subjected to a Western blot using anti-MERS-CoV S2 and anti-NDV HN antibodies. Recombinant S_MERS_ was used as a positive control. (**C**) The ALF and the UC-virus were measured for S_MERS_ in an anti-MERS-CoV S1 sandwich ELISA. (**D**) Viral RNAs of NDV-mCh and NDV-S_MERS_-S1/S2 were purified and subjected to RT-PCRs using S_MERS_- and N_NDV_-specific primers. (**E**) DF-1 cells were infected with NDV-S_MERS_-S1/S2 for 24-h and either fixed with ice-cold acetone, or formaldehyde. Cells were stained with mouse anti-MERS-CoV S1, followed by AlexaFluor-488-conjugated goat anti-mouse IgGs. Images were recorded at 20× magnification under a fluorescence microscope. (**F**) Ultracentrifuged NDV-mCh, NDV-S_MERS_-S1/S2, and NDV-S_MERS_-S2’ were subjected to a Western blot analysis using anti-MERS-CoV S2 antibody, and (**G**) an anti-MERS-CoV S1 sandwich ELISA. (**H**) The ultracentrifuged NDV-S_MERS_-S2’ sample was stained with rabbit anti MERS-CoV S1 antibody, then 10-nm gold-conjugated goat anti-rabbit IgGs. Images were recorded under a TEM at 50,000× magnification.

To construct an NDV displaying an intact S_MERS_ ectodomain, we first inserted a chicken-codon optimized S_MERS_ sequence containing the S1/S2 cleavage site and 2P mutations, but without S2’ mutation (called S_MERS_-S1/S2) into NDV La Sota genome between *P* and *M* genes (Figure 2A). To facilitate an efficient incorporation of S_MERS_ onto the surface of NDV particles, transmembrane domain and cytoplasmic tail of S_MERS_ were replaced with that of NDV fusion (F) protein [21]. Tissue plasminogen activator (tPA) was also used instead of the native signal sequence to increase S_MERS_’s membrane transport [22]. Using a reverse genetics technique, the NDV carrying S_MERS_-S1/S2 (called NDV-S_MERS_-S1/S2) was successfully generated in BHK21/WI-2 co-cultured with DF-1 cells and subsequently amplified in embryonated chicken eggs. Subsequently, we were able to obtain allantoic fluid samples positive for NDV virus as indicated by positive hemagglutination unit (HAU) in a hemagglutination assay. Unfortunately, when measured by S1-specific sandwich ELISA or S2-specific Western blots, S_MERS_ was not detected in the allantoic fluid or the ultracentrifuged virus samples (Figure 2B,C).

To verify whether the *S_MERS_* gene insert was retained in the viral genome, RT-PCR was performed using primers specific for *S_MERS_* gene. A band corresponding to the *S_MERS_* gene was found from the viral genomic RNA samples (Figure 2D), indicating that the lack of S_MERS_ protein expression was not due to the loss of the gene insert. Immunofluorescence staining of NDV-S_MERS_-S1/S2-infected cells was then conducted to determine whether the S_MERS_ protein was expressed in host cells. As shown in Figure 2E, S_MERS_ was clearly detected by anti-MERS-CoV S1 antibody when the cells were fixed with ice-cold acetone, but not when fixed with formaldehyde. These data suggested that the inserted *S_MERS_* gene was expressed within the host cells, but, through undetermined mechanisms, it failed to be expressed on the cell surface.

Because there is another well-known furin cleavage site at S2’ position of S_MERS_, we then examined whether an additional mutation at S2’ cleavage site (called S_MERS_-S2’) would improve S_MERS_ surface expression. As shown in Figure 2F,G, NDV with S_MERS_-S2’ (called NDV-S_MERS_-S2’) became ELISA and Western blot positive for S_MERS_ detection. Further verification by anti-S_MERS_ immune-gold staining observed under a TEM also confirmed that S_MERS_-S2’ was expressed on the surface of NDV particles (Figure 2H).

**Figure 3:**
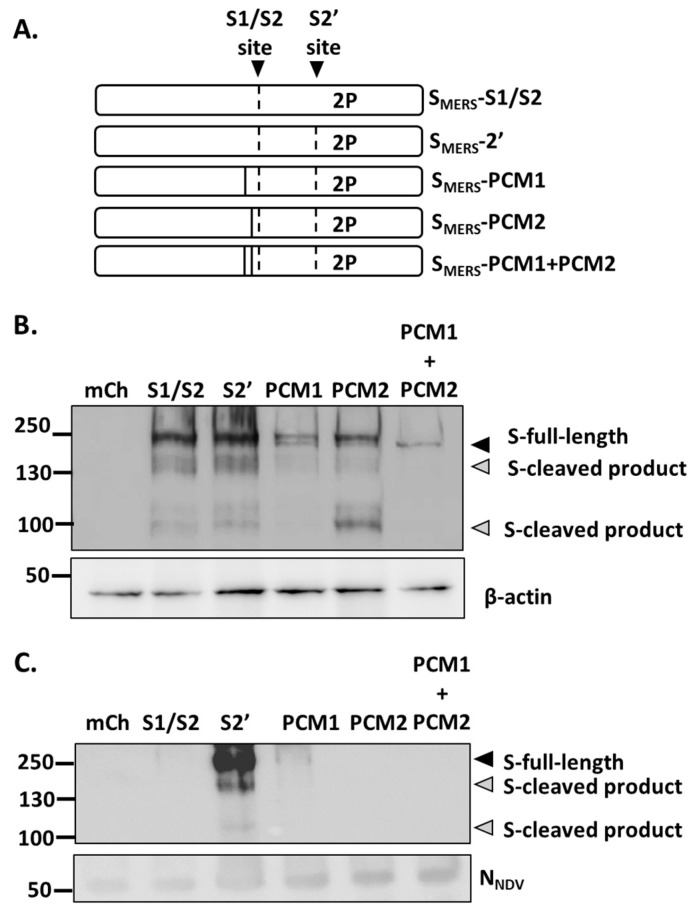
PCM1 and PCM2 did not improve S_MERS_ expression. (**A**) A diagram of different versions of S_MERS_ containing combinations of S1/S2, S2’, 2P, PCM1, PCM2 mutations. (**B**) HEK-293T/17 cells were transfected with 1 µg pCAGGS containing each version of *S_MERS_* gene. Cell lysates harvested at 72 h post transfection were subjected to a Western blot analysis using anti-MERS-CoV S2 and anti-β-actin antibodies. (**C**) The volumes of the ultracentrifuged virus samples were adjusted to equal TCID50/mL. The samples were then subjected to a Western blot analysis using anti-MERS-CoV S2 and anti-NDV N antibodies.

As seen in Figure 2F, a band corresponding to the full-length S_MERS_ of around 200 kDa was clearly observed from the NDV-S_MERS_-S2’ sample, however bands of lower molecular weights were also detected. We hypothesized that these lower bands were of cleaved S_MERS_ products. Using Prop-1.0 furin cleavage site prediction program (https://services.healthtech.dtu.dk/services/ProP-1.0/ (accessed on 7 November 2023)), two additional furin cleavage sites were predicted at positions 626-RQQR-629 and 691-RSTR-694. Previously Gierer et al. identified these exact mutations and named them as Potential Cleavage Mutation 1, “PCM1”, and Potential Cleavage Mutation 2, “PCM2”, respectively (Figure 3A) [23]. Gierer et al. also reported that Arg to Ala mutations at PCM1 together with S1/S2 site mutation prevented S_MERS_ from being cleaved, and that Arg to Ala mutations at PCM2 unexpectedly reduced the protein expression to an undetectable level.

The effects of PCM1 and PCM2 mutations were examined herein. Adding on to our codon-optimized S_MERS_-S2’already containing S1/S2, S2’ and 2P mutations (Supplementary Figure S1), *S_MERS_* genes with the additional PCM1 and/or PCM2 were transfect into HEK-293T/17 cells. As shown in Figure 3B, PCM1 appeared to diminish the cleaved S_MERS_ products. On the other hand, the cleaved products were visible in S_MERS_-PCM2, but of lower expression levels than that of S_MERS_-S1/S2 and S_MERS_-S2’. The combination of PCM1 and PCM2 further reduced S_MERS_ expression to the lowest.

These mutated *S_MERS_* genes were also used to generate NDV-S_MERS_ (Supplementary Table S1). The resulting viruses at comparable amounts (equal TCID_50_) were then analyzed on a Western blot. It was observed that only faint S_MERS_ protein bands were apparent in the NDV-S_MERS_-PCM1 sample, while NDV-S_MERS_-PCM2 and NDV-S_MERS_-PCM1 + PCM2 did not result in any visible S_MERS_ protein bands (Figure 3C). Collectively, these data suggested that PCM1 and/or PCM2 mutations did not improve S_MERS_ surface expression. NDV-S_MERS_-S2’ remained the best construct that displayed the highest amount of S_MERS_.

### 3.3. 2P Mutation Was Necessary for an Optimal S_MERS_ Surface Expression

**Figure 4:**
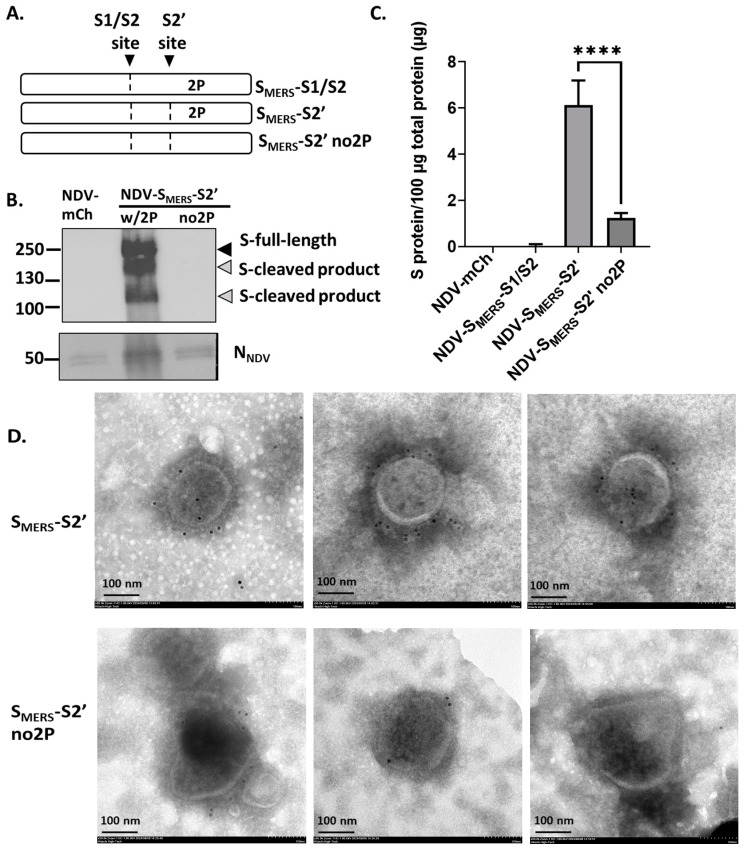
2P mutation improved surface S_MERS_ expression. (**A**) A diagram comparing the gene insert of NDV-S_MERS_-S1/S2, NDV-S_MERS_-S2’, and NDV-S_MERS_-S2’ no2P. (**B**) The ultracentrifuged virus samples were subjected to Western blot analysis using anti-MERS-CoV S2 and anti-NDV N antibodies, and (**C**) an MERS-CoV S1-sandwich ELISA. **** indicates significant difference (*p* < 0.0001). (**D**) The ultracentrifuged samples were stained with rabbit anti MERS-CoV S1 antibody, and then the 10-nm gold-conjugated goat anti-rabbit IgGs. Images were recorded under a TEM at 50,000× magnification.

Pallesen et al. previously demonstrated that the 2P mutation facilitated the maintenance of S_MERS_’s prefusion form [8]. Herein, we investigated whether the 2P mutation was necessary for the expression of S_MERS_ on NDV particles. NDV-S_MERS_-S2’ with or without 2P mutation (Figure 4A) were generated and subsequently subjected to S_MERS_ detection. As shown in Figure 4B, no protein bands as measured on a Western blot were detected from NDV-S_MERS_-S2’ without 2P mutation, however, low levels of S_MERS_ were detected in a MERS-CoV S1 sandwich ELISA (Figure 4C). The level of S_MERS_ in NDV-S_MERS_-S2’ without 2P mutation was drastically reduced (about 5-fold) as compared to NDV-S_MERS_-S2’ with 2P mutation. The results from S1-immunogold staining also correlated with the ELISA results, showing that while viral particles of NDV-S_MERS_-S2’ with 2P mutation were consistently positive for S_MERS_ staining, portions of NDV-S_MERS_-S2’ without 2P mutation were negative for S_MERS_ (Figure 4D, right image of the lower panel). Collectively, these data suggested that 2P mutation improved the stability of S_MERS_ displayed on the surface of NDV particles.

### 3.4. NDV-S_MERS_-S2’ Effectively Induced Humoral and Cellular Immune Responses in Mice

Among all of the versions of NDV-S_MERS_ that were constructed (Supplementary Table S1), NDV-S_MERS_-S2’ displayed the highest amount S_MERS_. We then examined whether NDV-S_MERS_-S2’ could be used as a vaccine against MERS-CoV.

The NDV-S_MERS_-S2’ was prepared as a live vaccine or inactivated with binary ethylenimine (BEI) for mouse immunization. A low and a high dose of inactivated NDV-S_MERS_-S2’ contained 100 and 250 ng of S_MERS_/50 µL, respectively. NDV-mCherry containing an equal amount of virus as that of the high dose NDV-S_MERS_-S2’ (3.9 × 10^6^ TCID50/mL) was used as a control. The same titer of NDV-S_MERS_-S2’ was also used in the live-vaccine regimen. Recombinant S produced in-house at 250 ng/50 µL was also used for comparison. While the PBS control, NDV-mCherry, the recombinant S_MERS_, and inactivated-NDV-S_MERS_-S2’ were prepared as a mixture with Addavax adjuvant, live NDV-S_MERS_-S2’ was injected without any adjuvant.

**Figure 5:**
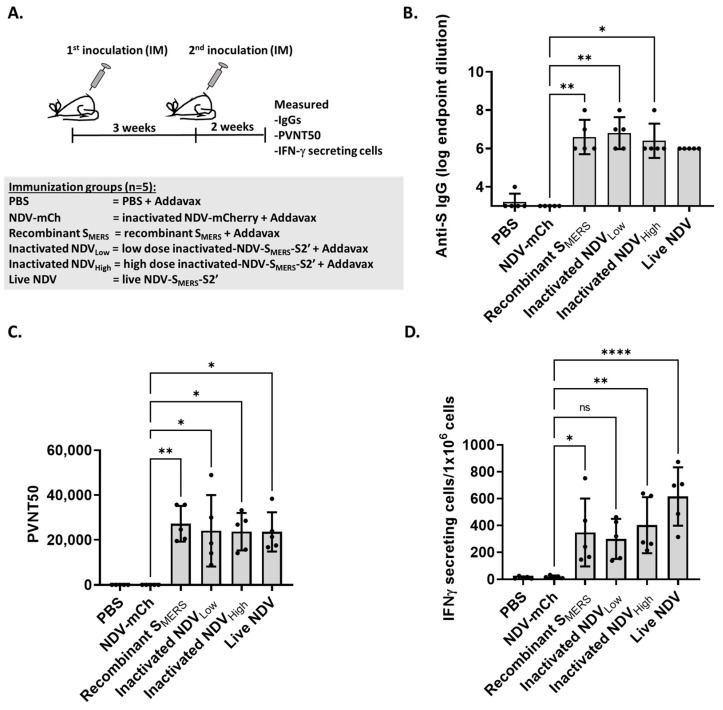
NDV-S_MERS_-S2’ induced strong humoral and cellular immune responses. (**A**) Groups of 5 mice were injected intramuscularly twice, 3 weeks apart with PBS + Addavax (PBS), inactivated NDV-mCherry + Addavax (NDV-mCh), recombinant S_MERS_, low dose inactivated-NDV-S_MERS_-S2’ + Addavax (Inactivated NDV_Low_), high dose inactivated-NDV-S_MERS_-S2’ + Addavax (Inactivated NDV_High_), or live NDV-S_MERS_-S2’. Two weeks after the second immunization, sera were collected and (**B**) anti-S_MERS_ IgGs were measured with anti-S_MERS_ IgG indirect ELISA. The last dilutions of sera that gave signals above average + 3x standard deviation of PBS wells are depicted. (**C**) Mouse sera were subjected to a pseudotype virus neutralization assay (PVNT50). Serum dilutions that yielded 50% of signals from cells transduced with pseudotyped virus alone was calculated using GraphPad Prism 9.0.0 (121). (**D**) Splenocytes were restimulated with S_MERS_ overlapping peptides for 24 h on IFN-γ ELISPOT plates. Spots representing numbers of IFN-γ secreting cells were developed and then counted using ImmunoSpot software 7.0.9.4 Professional Analyzer DC on a CTL ImmunoSpot S6 Universal Analyzer. Means ± S.D. are presented. One-way ANOVA with Tukey’s multiple comparison post-test was used to calculate significant differences between NDV-mCh and NDV-S_MERS_-S2’ immunized groups. *, **, **** indicate significant difference *p* < 0.05, *p* < 0.01, and *p* < 0.0001, respectively. ns indicates no significant difference.

BALB/cAJc-1 mice (*n* = 5) were injected intramuscularly twice, three weeks apart, with 50 µL of each vaccine regimen. Two weeks following the second immunization, mouse blood and splenocytes were collected for measurements of humoral and cellular immune responses (Figure 5A). As shown in Figure 5B, mice immunized with either a low or a high dose of inactivated NDV-S_MERS_-S2’ exhibited strong IgG titers against S_MERS_ protein. Similar levels of S_MERS_-specific IgGs were also noted in mice vaccinated with the live vaccine.

Neutralizing antibody levels were also measured using a lentiviral pseudotyped virus-based assay. This assay was performed using HEK-293T cells engineered in-house to express the MERS-CoV receptor, dipeptidyl peptidase-4 (DPP4 or DPPIV), also known as adenosine deaminase complexing protein 2 or CD26. Mice immunized either with recombinant S_MERS_, a low or a high dose of inactivated vaccine, and live vaccine, all had similarly high levels of PVNT-50 titers of about 3 × 10^4^.

The difference in host immune responses among vaccinated groups was observed in cellular immune responses as measured by IFN-γ ELISPOT. Among all vaccine regimens tested, the live virus vaccine was noted as the strongest inducer of IFN-γ producing cells. Overall, our data demonstrated that both inactivated and live NDV-S_MERS_-S2’ effectively induced humoral immune responses. The live virus vaccine, as expected, performed the best as an inducer of cellular immune responses.

### 3.5. S_MERS_ Insert Was Stable After Virus Passaging in Embryonated Chicken Eggs

**Figure 6:**
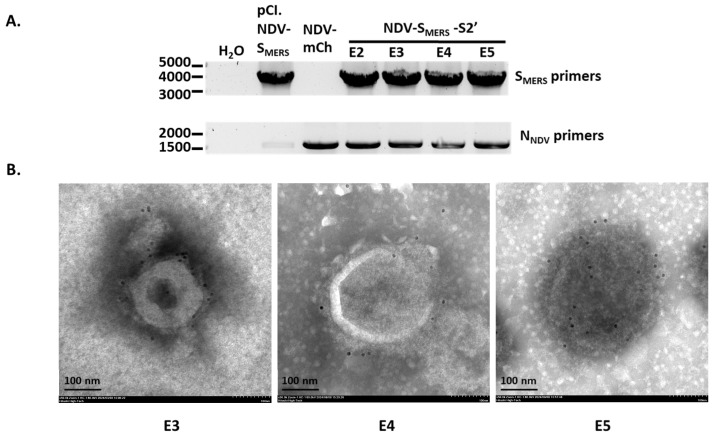
*S_MERS_* gene insert was stable after 5 egg-passages. (**A**) Viral RNAs of NDV-mCh and NDV-S_MERS_-S2’ from egg-passages 2–5 were purified and subjected to RT-PCRs using S_MERS_- and N_NDV_-specific primers. pCI.NDV-S_MERS_ plasmid was used as positive controls. (**B**) The ultracentrifuged NDV-S_MERS_-S2’ samples from egg-passage 3–5 (E3, E4, and E5) were stained with rabbit anti MERS-CoV S1 antibody, followed by 10-nm gold-conjugated goat anti-rabbit IgGs. Images were recorded under a TEM at 50,000× magnification.

One of the desired features for a viral vector-based vaccine is its ability to maintain antigenic gene insert. We examined whether the *S_MERS_* gene insert was retained in the viral genome after 5 egg-passages. Viral RNAs were extracted and subsequently subjected to RT-PCR using primers specific to the *S_MERS_* gene. As shown in Figure 6A, *S_MERS_* gene was consistently detected in the E2-E5 NDV-S_MERS_-S2’ genome. Anti-S1 immunogold staining also confirmed the presence of S_MERS_ on the particles of E3-E5 NDV-S_MERS_-S2’. These data demonstrated the stability of gene insertion within the NDV genome throughout the egg-passages, suggesting the suitability of NDV-based vector for recombinant vaccine development.

## 4. Discussion

MERS-CoV is a virus that causes severe respiratory disease with a high mortality rate. Effective vaccines are still needed specially for populations at risk, such as those handling dromedary camels. In this study, we demonstrated that NDV, both in an inactivated and a live vaccine format, is a suitable and effective vaccine platform for inducing host immune responses against MERS-CoV. Unlike a previous study that examined NDV carrying an unmodified viral sequence of S_MERS_ [4], our study exploited a codon-optimized, 2P-mutated S_MERS_ as an antigen. We also showed that the mutations at S1/S2 and S2’ cleavage sites were necessary for surface expression of S_MERS_ on NDV particles. Moreover, further mutations at PCM1 and PCM2 sites did not improve S_MERS_ expression.

The first design of our NDV-S_MERS_ vaccine was composed of S_MERS_ containing S1/S2 and 2P mutations. This combination of mutations was previously shown to sufficiently stabilize recombinant S_MERS_ ectodomain in its prefusion form [8]. However, our experiments demonstrated that the two mutations, when in the context of NDV-S_MERS_ construct, were not sufficient to mediate surface expression of S_MERS_ in infected cells (Figure 2E, formaldehyde-fixed cells) or on NDV particles (Figure 2B,C). We suspected that the S_MERS_-S1/S2 was still sensitive to proteases present in allantoic fluid [24] or to trypsin used in NDV propagation in cell culture. We attempted to detect the residual cleaved S_MERS_ products in the allantoic fluid or in NDV-S_MERS_-S1/S2 infected culture supernatants, however, no S_MERS_ was detected by the anti-MERS-CoV S1, or anti-MERS-CoV S2 antibodies used. It is possible that the cleaved S_MERS_ protein was already degraded, or the conformation of the cleaved products was no longer recognized by the antibodies. Further examinations are needed to elucidate the fate of these cleaved S_MERS_ products.

The inability of NDV-S_MERS_-S1/S2 to express S_MERS_ on the surface was resolved by adding a S2’ mutation to the construct. Although significant levels of S_MERS_ were detected on the NDV-S_MERS_-S2’ virus by a sandwich ELISA (Figure 2G) and immunogold staining (Figure 2H), Western blot analyses consistently revealed 3 distinct bands at the sizes of around 200, >130, and >100 kDa. The 200 kDa band is likely to be of the full-length uncleaved S_MERS_, while the >130 KDa size was proposed to be from the cleavage at PCM1 position [25]. Since MERS-CoV S2 domain was reported to be around 100 kDa [23,25], a product at >100 kDa is possibly derived from a cleavage at PCM2, located on the N-terminal side of S1/S2 cleavage site [19]. However, we showed that the >100 kDa was still visible even when the PCM2 was mutated (Figure 3B). Thus, another nearby proteolytic cleavage site may exist. Using Expasy PeptideCutter program (web.expasy.org (accessed on 18 July 2023)) to analyze the S_MERS_ amino acid sequence, it was revealed that amino acid positions 698, 699, 700, 728 may be vulnerable to trypsin cleavage. Because the identification of extra S_MERS_ cleavage sites may help improve S_MERS_ presentation on NDV particles, we are continuing our investigation to pinpoint the precise cleavage site responsible for producing the >100 kDa product.

Although NDV-S_MERS_-S2’ displayed cleaved S_MERS_ products by Western blot, a significant amount of uncleaved S_MERS_ was still distinctly detected. We showed that the 2P mutation also contributed to the stability of S_MERS_ on NDV particles. In the absence of 2P mutation, the surface S_MERS_ was detected, but at a considerably reduced amount (Figure 4B,D). The 2P mutation-mediated restraint of S_MERS_ is crucial particularly when S_MERS_ is expressed on NDV particles. Because allantoic fluid is reported to contain DPP4 and is rich in proteases [24], S_MERS_, when in this environment, can bind to DPP4, consequently changing its conformation and exposing its buried cleavage sites to proteases. The 2P mutation likely helps prevent these conformational changes and stabilizes the protein on NDV particles.

In this study, we initially aimed to develop an inactivated NDV-based MERS vaccine. Thus, it was essential to have the S_MERS_ ectodomain displayed on the surface of NDV particles. However, the NDV platform has also been used effectively in live virus vaccines [5,21]. Data presented in Figure 5 clearly show that both inactivated and live vaccine formats of NDV-S_MERS_-S2’ elicited robust humoral immune responses in mice, with PVNT50 titers reaching as high as 3 × 10^4^. This is in contrast to the results from a previous study using an NDV carrying the native S_MERS_ sequence, which showed neutralizing antibody titer of about 2^8^ as measured by a VSV psuedotype-based neutralization assay [4]. Although our PVNT50 assay cannot be directly compared to their neutralization assay, the high PVNT50 titer observed in this study indicates that NDV-S_MERS_-S2’ is a highly effective inducer of neutralizing antibodies. This suggests that the codon optimization and S_MERS_ mutations described here significantly improved the antigenicity of S_MERS_.

Regarding the cellular immune responses, a notable higher number of IFN-γ-secreting cells was observed in live virus-vaccinated group as compared to the inactivated vaccine groups. This result was unsurprising as live vaccines can infect host cells [26], thereby efficiently providing intracellular antigens for T cell stimulation. While inactivated vaccines are generally safe for all populations, including the elderly and immunocompromised individuals, live NDV vaccines could provide enhanced cellular immune responses and may offer a more cost-effective production option. Depending on the vaccination goals, both inactivated and live NDV-based MERS vaccines represent promising, safe, and effective choices for MERS immunization.

The NDV-based MERS vaccine described herein has demonstrated effectiveness in inducing strong host immune responses against SMERS. Although we were unable to evaluate the NDV-SMERS-S2’ vaccine in a MERS-CoV challenge model due to a lack of wildtype MERS-CoV isolates and an animal biosafety level-3 (ABSL-3) facility, the promising immune responses observed underscore the vaccine’s potential.

In comparison to other viral vector vaccine platforms, such as adenoviral vectors, NDV is a platform that can be easily manipulated. In our NDV-rescue system, only one-step cloning of the NDV genome is required to replace the inserted antigen. Furthermore, NDV can be grown in embryonic chicken eggs to high titers, providing a cost-effective platform for vaccine production. More importantly, the NDV-based MERS vaccine may also be further developed for use as an intranasal vaccine, given its ability to infect cells of the respiratory tract [27]. Moreover, the very low levels of pre-existing anti-NDV immunity in the human population minimizes the risk of anti-vector immune interference [28]. NDV infection also induces the production of type I interferons, which can act as natural adjuvants, stimulating various arms of the host immune response [29]. Recent studies of an intranasal NDV-based vaccine carrying the spike protein of SARS-CoV-2 have demonstrated that NDV can safely and effectively induce host immune responses [5,30]. These findings highlight the potential of NDV as an effective vector for MERS.

In summary, we demonstrated in this study that our NDV-S_MERS_-S2’ vaccine elicited robust humoral and cellular immune responses specifically to the spike protein of MERS-CoV. Moreover, the sera of immunized mice displayed strong neutralizing activity against the MERS-CoV pseudotyped lentivirus. Future studies incorporating a challenge model will be crucial to fully assess the potency of the NDV-S_MERS_-S2’ vaccine and advancing its development.

## 5. Conclusions

The NDV-based vaccine platform is a safe and promising technology for vaccine development. This study demonstrates that modifications of the S_MERS_ sequence, including codon optimization, S1/S2, S2’, and 2P mutations, effectively preserved S_MERS_ on NDV particles. The resulting NDV-S_MERS_-S2’ virus efficiently elicited strong humoral and cellular immune responses, underscoring the potential of the NDV vector as a robust platform for MERS vaccine development.

## Figures and Tables

**Table 1:** Sequence analyses for codon usage preference in host cells.

Host Cells	S_MERS_ Sequences	CAI ^a^	GC3 ^b^	CPS ^c^
Mammalian cells (BHK-21/WI-2)	Viral sequence	0.63	0.32	0.10
	Mammalian-codon optimized	0.76	0.58	−0.10
	Chicken-codon optimized	0.79	0.66	−0.06
Chicken cells (DF-1)	Viral sequence	0.75	0.32	0.01
	Mammalian-codon optimized	0.83	0.58	−0.09
	Chicken-codon optimized	0.84	0.66	−0.02

^a^ codon adaptation index; ^b^ GC content at the third position of synonymous codons; ^c^ codon pair scores.

## Data Availability

Correspondence and requests for additional materials and data should be addressed to N.W.

## Supplementary Materials

The following supporting information can be downloaded at: https://www.mdpi.com/article/10.3390/vaccines13010002/s1, Figure S1: Diagram showing S_MERS_ with potential cleavage and proline substitution sites; Table S1: Various NDV-SMERS with the modifications described in this study.
