# Supplementary material for: Newcastle Disease Virus Displaying an Ectodomain of Middle East Respiratory Syndrome Coronavirus Spike Protein Elicited Robust Humoral and Cellular Immunity in Mice
Source: Vaccines (Basel). 2024 Dec 24;13(1):2. doi: 10.3390/vaccines13010002 (PMC11768598; doi:10.3390/vaccines13010002)
Supplement: Supplementary file 1 [file vaccines-13-00002-s001.zip › vaccines-3219068-supplementary.pdf]

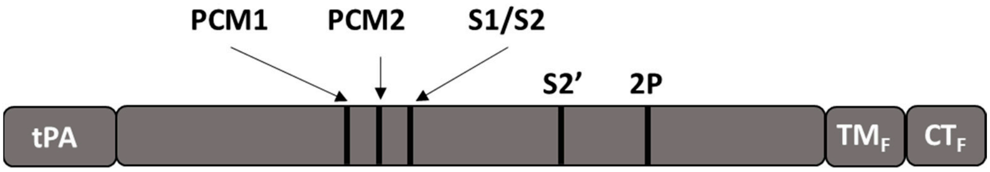

Positions	Original Sequences	Mutated Sequences
**PCM1**	626-RQQR-629	626-AQQA-629
**PCM2**	691-RSTR-694	691-ASTA-694
**S1/S2**	748-RSVR-751	748-ASVG-751
**S2’**	884-RSAR-887	884-GSAG-887
**2P**	V1060, L1061	P1060, P1061

Figure S1. Diagram showing S_{MERS} with potential cleavage and proline substitution sites.

Virus Names	S_{MERS} mutations				
	S1/S2	S2’	PCM1	PCM2	2P
**NDV-S_{MERS}-S1/S2**	✓				✓
**NDV-S_{MERS}-S2’**	✓	✓			✓
**NDV-S_{MERS}-S2’ no2P**	✓	✓			
**NDV-S_{MERS}-PCM1**	✓	✓	✓		✓
**NDV-S_{MERS}-PCM2**	✓	✓		✓	✓
**NDV-S_{MERS}-PCM1+PCM2**	✓	✓	✓	✓	✓

Table S1. Various NDV-SMERS with the modifications described in this study.
